# Trends and determinants of breastfeeding within one hour in Ethiopia, further analysis of Ethiopian Demographic and Health Survey: multivariate decomposition analysis

**DOI:** 10.1186/s13052-021-01032-5

**Published:** 2021-03-26

**Authors:** Tilahun Yemanu Birhan, Wullo Sisay Seretew, Muluneh Alene

**Affiliations:** 1 Department of Epidemiology and Biostatistics, Institute of public health, College of Medicine and Health Science, University of Gondar, Gondar, Ethiopia; 2 Department of Public Health, College of Health Science, DebreMarkos University, Debre Markos, Ethiopia

**Keywords:** Breastfeeding, Ethiopia, Multivariate decomposition analysis and trend

## Abstract

**Background:**

Despite the substantial efforts to improve timely/early initiation of breastfeeding, avoidance of colostrum, and delayed initiation of breastfeeding remains a big challenge in developing countries. Therefore, this study aimed to analyze the trends of early breastfeeding rate over time based on the Ethiopian Demographic and Health Survey (EDHS).

**Methods:**

Secondary data analysis was conducted based on the Ethiopian Demographic Health Surveys (EDHSs) conducted in 2005, 2011, and 2016. A total weighted sample of 9, 111, 10,106, and 8564 in 2005, 2011, and 2016 respectively were included for analysis. Trend and Logistic based decomposition analysis technique was used for analyzing the trends of early breastfeeding initiation over time and factors contributing to the change in early breastfeeding initiation rate. STATA 15 was employed for data management and analyses. All analyses presented in this paper were weighted for the sampling probabilities and non-response.

**Result:**

Among children age less than 5 years the rate of early breastfeeding initiation rate overtime was increased from 70.5% in 2005 to 72.7% in 2016. The highest rate of improvement was seen in the second phase of the study (2011–2016) while it shows a decline in the first phase (2005–2011) from 70.5 to 55.1%. The decomposition analysis indicated that about half of the overall change in early breastfeeding initiation rate was due to the difference in women’s composition. Particularly, an increase in health facility delivery and vaginal delivery was a significant predictor of the increasing rate of early breastfeeding initiation over the surveys.

**Conclusion:**

Early initiation of breastfeeding slightly increasing over the last 10 years in Ethiopia. Half of the overall increase in the early initiation of breastfeeding was due to the change in compositional characteristics of women over 10 years in Ethiopia. Change in the composition of women according to health facility delivery and vaginal delivery were the major source of the increase in early breastfeeding initiation over time. Public interventions including promoting health facility delivery of women for further improvements of early breastfeeding initiation should be needed.

## Introduction

Early initiation of breastfeeding after birth is an essential intervention to reduce neonatal mortality and morbidity [1–3]. Despite the substantial efforts to improve timely/early initiation of breastfeeding, avoidance of colostrum, and delayed initiation of breastfeeding remains a big challenge in developing countries [1, 4, 5]. The World Health Organization (WHO) early initiation of breastfeeding within 1 hour as it confers many benefits to the child and prevents neonatal mortality that extends into adulthood [6, 7]. Early initiation of breastfeeding stimulates the production of breast milk and ensures greater consumption of highly nutritious colostrum breast milk produced during the first few days after birth [1, 8, 9]. Early initiation of breastfeeding also an important bridge for the mother-to-child relationship and to increase the length of breastfeeding [10–12]. In addition to these advantages, early initiation of breastfeeding induces uterine contraction after pregnancy, decreasing the risk of postpartum haemorrhage and extending the duration of postpartum infertility, allowing women to return to their presentational weight, as well as reducing the risk of breast and ovarian cancer [9, 11, 13]. Globally, the prevalence of early initiation of breastfeeding ranged from 17.7 to 98.4% with an average of 57.6%, a slightly lower prevalence among mothers with complications during pregnancy and caesarean delivery [5].

Globally, an estimated 4 million newborns die each year from preventable infectious disease, which accounts 41% of mortality among under-five children [14]. In addition, most of the deaths occur within the first week of life, which is considered to be the early neonatal period [15]. The risk of newborn death during the first day of life was approximately 1 % globally, of which two-thirds of the burden already exists in ten countries, including Ethiopia [2, 16]. In recent decades, progress towards improving early initiation of breastfeeding has been sluggish, with the average rate of global initiation only increasing by 14%age points. However, less than half of all newborns are placed to the breast within an hour of birth globally. In the least developed region, the percentage of early initiation of breastfeeding was 53% [17], while the total pooled prevalence of early initiation of breastfeeding in Ethiopia was 61.4% [2]. Different studies on determinants of early initiation of breastfeeding showed that rural residence, educational status, mode of delivery, use of antenatal care (ANC), marital status, and place of delivery were important predictors of early initiation of breastfeeding [2, 3, 18–21]. Only point survey data was analyze in previous studies [11, 20–26]; it was difficult to see the patterns and to identify potential factors that have been consistently influenced early breastfeeding initiation over time in Ethiopia.

Studying the change in early initiation breastfeeding using multivariate decomposition analysis to identify predictors associated with the change in early initiation breastfeeding overtime becomes relevant for targeting interventions to improve early initiation of breastfeeding rate over time and could critically inform policies and strategies aimed to rise early initiation of breastfeeding rate in Ethiopia. Therefore, this study aimed to evaluate trends and determinants of early initiation of breastfeeding rate in Ethiopia.

## Methods and materials

### Data

This study was conducted on secondary data analysis obtained from 2005, 2011, and 2016 Ethiopian Demographic and Health Surveys (EDHS). A stratified two-stage cluster sampling technique was used by the EDHS, chosen as a sampling frame in two stages using the 1994 Population and Housing Census (PHC) framework for EDHS 2005 and the 2007 Population Census (PHC) framework for EDHS 2011 and 2016. Stratification was maintained by separating each region into urban and rural areas. Twenty-one sampling strata were established because the area of Addis Ababa was entirely urban. In the first phase, 540 Enumeration Areas (EAs) (145 in urban areas) were selected for EDHS 2005, 624 EAs (187 in urban areas) were selected for EDHS 2011, and 645 EAs (202 in urban areas) were selected for EDHS 2016 by probability proportional to the size of the enumeration region and independent selection of each sampling stratum. At the second stage, a complete household listing operation was carried out in all selected EAs prior to the start of the fieldwork and an average of 28 households were selected systematically. The detailed sampling strategies are fully presented in the EDHS report [27–29]. The source population is all under-five children in Ethiopia, whereas the study population was all underfive children in the selected enumeration region.

### Outcome variable

The outcome variable was early initiation of breastfeeding practice (EIBF) among mothers with children younger than 24 months of age. The initiation of breastfeeding within 1 h of birth is an early initiation of breastfeeding. Initiating breastfeeding was coded as ‘1’ within 1 h of birth, while beginning breastfeeding was coded as ‘0’ for logistic regression analysis after 1 h of birth.

### Independent variables

Socio-demographic and economic variables (residence, region, maternal age, marital status, religion, maternal education, paternal education, wealth index, maternal occupation, maternal working Status), Pregnancy and pregnancy-related factors (ANC visit, Parity, Preceding birth interval, contraceptive use, Place of delivery, Birth order, Mode of delivery, wanted pregnancy). Behavioural factors.

(Smoking, media exposure) were included in this study.

### Data collection procedure

The study was conducted based on EDHS data by accessing from the DHS program official database www.measuredhs.com after permission was granted through an online request by explaining the objective of our study. The raw data was collected from all parts of the country on childbearing aged women using a structured and pre-tested questionnaire. We used the kid Record (KR file) data set and extracted the outcome and independent variables.

### Data management and analysis

Before any statistical analysis, the data were weighted using sampling weight, primary sampling unit and strata to restore the representativeness of the survey and to inform STATA to take account of the sampling design when calculating standard errors to obtain reliable parameter estimates. Since the response variable have binary outcome, binary logistic regression model was employed. Sampling was applied as part of a complex survey design using primary sampling unit, strata, and women’s individual weight (V005). Data from 2005, 2011, and 2016 were appended together after extracting relevant variables for trend and decomposition analysis. Cross tabulation and summary statistics were performed to describe the study population using STATA 14 software.

### Trend and decomposition analysis

The trend was assessed using descriptive statistics stratified by selected background characteristics of respondents and was assessed separately in the periods 2005–2011, 2011–2016, and 2005–2016. A multivariate analysis of the shift in the early initiation of breastfeeding rate was employed to address possible factors contributing to the difference in the percentage of early initiation of breastfeeding over the study era. This approach is used for many purposes in economics, demography, medicine, and other specialties. This research focused on how early initiation breastfeeding rate reacts to the variation in women’s characteristics and how these variables form the difference across surveys conducted at different times. The study was a regression analysis of the difference in the percentage of early initiation of breastfeeding rate between EDHS 2005 and 2016. Decomposition analysis aimed to determine the cause of the discrepancy in the percentage of EIBF in the last 10 years. Both the difference in composition (Endowment) of the population and difference in the effect of characteristics (Coefficients) between the surveys is necessary to know the factors contributing to the increase/decrease in the EIBF rate over time. The multivariate decomposition analysis for the nonlinear response model utilizes the output from the logistic regression model since it is “a binary outcome” to parcel out the observed difference in the EIBF rate between the surveys into components. The difference in the rate of EIBF between the surveys can be attributed to the compositional difference in population (difference in characteristics or endowment) and the difference in the effect of explanatory variables (difference in coefficients) between the surveys.

Logit based decomposition analysis technique was used for the analysis of factors contributing to the change in EIBF over time to identify factors contributing to the change in the EIBF rate in the last 10 years. The variations of EIBF over time can be due to the compositional disparity between surveys and discrepancies in the results of the chosen explanatory. Hence, the observed variation in EIBF between surveys is additively decomposed into a characteristics (or endowments) component and a coefficient (or effects of characteristics) component. For logistic regression, the Logit or log-odd of EIBF is taken as:

$$ Logit(A)- Logit(B)=F\left({X}_A{\beta}_A\right)-F\left({X}_B{\beta}_B\right)=\underset{E}{\underbrace{\left[\mathrm{F}\left(\mathrm{XA}\upbeta \mathrm{A}\right)-\mathrm{F}\left(\mathrm{XB}\upbeta \mathrm{A}\right)\right]}}+\underset{C}{\underbrace{\left[\mathrm{F}\left(\mathrm{XB}\upbeta \mathrm{A}\right)-\mathrm{F}\right(\mathrm{XB}\upbeta \mathrm{B}\Big]}} $$

The *E* component refers to the part of the differential owing to differences in endowments or characteristics. The *C* component refers to that part of the differential attributable to differences in coefficients or effects [30].

The equation can be presented as:

$$ \mathrm{Logit}\ \left(\mathrm{A}\right)-\mathrm{Logit}\ \left(\mathrm{B}\right)=\left[\upbeta 0\mathrm{A}-\upbeta 0\mathrm{B}\right]+\Sigma \mathrm{XijB}\ast \left[\upbeta \mathrm{ijA}-\upbeta \mathrm{ijB}\right]+\Sigma \upbeta \mathrm{ijB}\ast \left[\mathrm{XijA}-\mathrm{XijB}\right] $$

- *X*_*ij*B_ is the proportion of the j^th^ category of the i^th^ determinant in the DHS 2005,
- *X*_*ij*A_ is the proportion of the j^th^ category of the i^th^ determinant in DHS 2016,
- *β*_*ijB*_ is the coefficient of the j^th^ category of the i^th^ determinant in DHS 2005,
- *β*_*ij*A_ is the coefficient of the j^th^ category of the i^th^ determinant in DHS 2016,
- *β*_0B_ is the intercept in the regression equation fitted to DHS 2005, and
- *β*_*0A*_ is the intercept in the regression equation fitted to DHS 2016

The recently developed multivariate decomposition for the non-linear model was used for the decomposition analysis of early initiation of breastfeeding using the mvdcmp STATA command [30]. In this study, variables with *p*-value < 0.2 in the bivariable multivariate decomposition analysis were considered for the multivariable multivariate decomposition analysis. In the multivariable multivariate decomposition analysis variables with *p*-value < 0.05 in the endowments and coefficients were considered as a significant contributing factor for the change in early initiation of breastfeeding over time.

## Result

### Characteristics of the study population

In the age group of fewer than 20 years, nearly three-fourths of respondents were found in all three surveys. Based on ANC visits, the number of ANC visits in the first quarter increased from 29% in 2005 to 37% in 2016. With regard to maternal education, 76% of women were not taught in 2005, compared to 65% in 2016. In addition, the percentage of primary education for husbands and spouses grew from 26% in 2005 to 36% in 2011, but decreased marginally to 33% in 2016 (Table 1).

**Table 1:** Percentage distribution of socio-demographic characteristics among respondents, 2005, 2011 and 2016 Ethiopian Demographic and Health Survey

Characteristics		Weighted frequency(%) 2005 *N* = 9111	Weighted frequency(%) 2011 *N* = 10,106	Weighted frequency(%) 2016 *N* = 8564
Trimester of ANC visit	1st trimester	29.29	29.95	37.29
	2nd trimester	52.71	55.74	53.22
	3rd trimester	18.00	14.32	9.49
Region	Tigray	10.12	10.47	8.89
	Afar	5.77	10.46	10.64
	Amhara	15.05	11.82	9.26
	Oromia	20.00	15.21	15.85
	Somali	6.54	9.21	14.37
	Benishangul-Gumuz	6.06	8.15	8.14
	SNNPR	18.01	13.85	12.46
	Gambela	5.21	7.23	6.45
	Harari	5.12	5.31	6.20
	Addis-Abeba	3.86	2.26	4.10
	Dire-Dawa	4.32	6.02	4.66
Religion	Orthodox	40.97	30.04	28.06
	Protestant	18.55	20.08	18.71
	Muslim	39.52	48.91	52.51
	Catholic	0.95	1.00	0.72
Husband educational status	None	59.42	55.73	50.06
	Primary	26.26	36.29	32.83
	Secondary+	14.32	8.00	17.12
women educational status	None	76.85	75.17	65.00
	Primary	15.74	23.00	25.12
	Secondary+	7.41	1.85	9.93
Wealth Index	Poor	43.35	51.48	55.36
	Middle	18.92	17.01	13.88
	Rich	37.72	31.51	30.76
Residence	Rural	86.00	86.65	83.10
	Urban	14.00	13.35	16.90
Place of delivery	Home	94.7	89.82	73.30
	H institution	5.3	10.17	26.70
Media exposure on EIBF	Exposed	37.28	58.67	68.17
	Non-exposed	62.71	41.03	32.83
Sex of child	Male	51.00	51.32	51.89
	Female	49.00	48.68	48.11
Birth order	1	19.48	15.42	18.94
	2–3	31.38	27.75	31.53
	4–5	22.70	20.64	23.94
	6+	26.44	36.20	25.60
ANC Visit	No	43.68	38.57	24.09
	Yes	56.32	61.43	75.91
Parity	1	12.49	9.43	12.56
	2–3	32.20	28.06	31.62
	4–5	24.43	22.44	25.55
	6+	30.88	40.07	30.27
Marital status	Single	11.63	23.32	45.1
	Married	88.37	76.71	5.0
Family size	< 5	68.38	64.39	76.23
	≥ 5	31.62	35.61	23.77
Mothers age at birth	< 20	74.10	73.23	72.68
	20–34	25.80	26.60	27.14
	35–49	0.11	0.13	0.19
Delivery type	Vaginal	98.24	97.58	97.13
	CS	1.76	2.42	2.87
Birth Interval	< 24 months	18.63	28.80	23.31
	≥ 24 months	81.37	71.20	76.69

The media representation of respondents increased from 37% in 2005 to 68% in 2016, and the number of women without an ANC visit fell dramatically from 72% in 2005 to 24% in 2016. The proportion of respondents’ institutional delivery was increased from 5.8% in 2005 to 26% in 2016. All variables listed in the table show change in composition, when we compare the sample population in the years 2005, 2011, and 2016.

### Trends of early initiation of breastfeeding (EIBF)

**Fig. 1:**
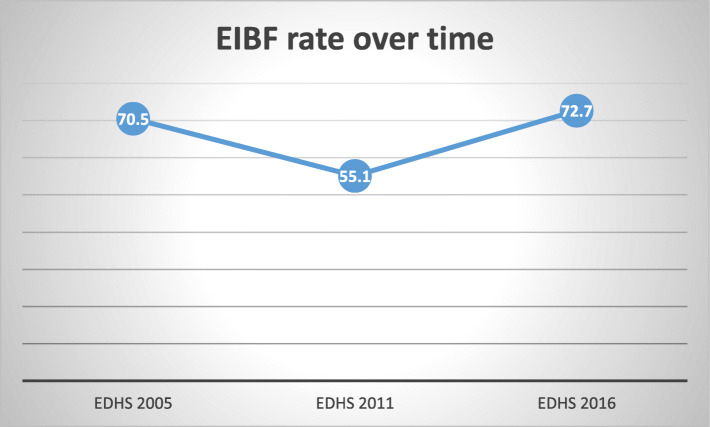
trends of breastfeeding within 1 hour in Ethiopia

The trend period was divided into three phases, 2005–2011, 2011–2016, and 2005–2016 in order to see the difference in early initiation of breastfeeding rate over time and the potential source for the change in the EIBF rate. The rate of EIBF over the study period (2005–2016) has been increased while it shows a decline from 2005 to 2011. The highest improvement was seen in the second phase (2011–2016) i.e. 17.6 percentage point changes in the EIBF rate but the rate of early initiation of breastfeeding was decreased from 70.55 to 55.1% in the first phase 2005–2011 (Fig. 1). The initiation of breastfeeding within the recommended time varies across background characteristics of the study population over the study period. The trends of breastfeeding rate within 1 hour over the study period differ across regions of Ethiopia. Initiation of breastfeeding within 1 hour was improved in the Tigray region in each phase i.e. 5.4% point change from 2005 to 2011, 8.3% from 2011 to 2016, and an overall change of 10% points in 2005–2016. However, initiation of breastfeeding within 1 hour was a decline in the Afar region over the study period by 48% points from 2005 to 2016. Besides, there was an increase in initiation of breastfeeding rate within 1 hour among women with secondary and higher education, an increase in the third phase of the study period (2005–2016) by 11 percentage points. Also, there was an increase in initiation of breastfeeding within 1 hour among women delivered at health institution such as in the second phase (2011–2016) it increases by 25 percentage points and in the third phase (2005–2016) increases by 16 percentage points (Table 2).

**Table 2:** Trends of early initiation of Breastfeeding in Ethiopia by selected characteristics 2005, 2011, and 2016 Ethiopian Demographic and Health Survey

Characteristics	2005 *N* = 9111	2011 *N* = 10,106	2016 *N* = 8564	The percentage point difference for EIBF		
				Phase I 2005–2011	Phase II 2011–2016	Phase III 2005–2016
**Age of women at 1st birth**						
< 20	70.06	54.64	71.95	−15.42	17.31	1.89
20–34	71.70	53.80	74.37	−17.90	20.57	2.67
35–49	75.10	44.44	90.00	−30.66	45.56	14.90
**Region**						
Tigray	51.57	53.25	61.55	5.43	8.30	9.98
Afar	89.27	57.72	41.63	−32.00	−16.09	−47.64
Amhara	62.53	54.33	69.18	−8.20	14.85	6.65
Oromia	72.16	56.49	79.41	−15.67	22.82	7.25
Somali	91.16	51.95	79.16	−39.21	27.21	−12.00
Benishangul-Gumuz	67.62	58.15	71.75	−9.47	13.60	4.13
SNNPR	70.82	51.52	79.11	−19.30	27.59	8.29
Gambela	71.56	54.04	73.74	−17.52	19.70	2.18
Harari	72.35	46.37	92.62	−25.98	46.25	20.27
Addis-Abeba	63.90	59.56	69.89	−4.34	10.33	5.99
Dirie-Dawa	90.90	57.25	89.38	−33.65	32.13	−1.52
**Religion**						
Orthodox	63.91	55.04	68.52	−8.87	13.48	4.61
Protestant	76.78	52.63	83.33	−24.15	30.70	6.55
Muslim	72.50	54.41	76.47	−18.09	22.06	3.97
Catholic	76.96	54.08	73.67	−22.88	19.59	−3.29
**Husband/partner educational status**						
None	72.91	52.88	70.18	−20.03	17.30	− 2.73
Primary	67.66	57.16	74.84	−10.50	17.68	7.18
Secondary+	66.77	52.76	75.40	−14.01	22.64	8.63
**Women educational status**						
None	72.40	53.50	71.57	−18.90	18.08	−0.83
Primary	64.64	57.10	73.51	−7.54	16.41	8.87
Secondary+	64.84	53.49	76.26	−11.35	22.77	11.42
**Household Wealth quantile**						
Poor	73.73	53.24	68.15	−20.49	14.95	−5.58
Middle	69.13	57.33	76.36	−11.80	19.03	7.23
Rich	67.64	55.14	77.78	−12.50	22.77	10.14
**Residence**						
Rural	68.30	54.20	78.43	−14.10	24.23	10.13
Urban	70.91	55.38	71.26	−15.53	15.88	0.35
**Place of Delivery**						
Home	71.52	54.47	70.01	−17.05	15.54	−1.52
H institution	63.21	54.21	78.91	−9.00	24.70	15.70
**Birth order**						
**1**	65.63	53.78	68.25	−11.85	14.47	2.62
2–3	71.10	55.23	75.73	−15.87	20.50	4.63
4–5	71.13	54.20	72.98	−16.93	18.78	1.85
6+	72.44	54.05	71.95	−18.39	17.90	−0.49
**Trimester of ANC visit**						
1st trimester	63.52	57.78	73.77	−5.74	10.25	10.52
2nd trimester	60.98	55.02	73.56	−5.96	12.58	12.58
3rd trimester	66.10	52.10	68.15	−14.00	16.05	2.05
**Media exposure**						
Exposed	71.44	55.18	73.91	−16.26	18.73	2.47
Non-exposed	69.93	54.03	72.01	−15.90	17.98	2.08
**Parity**						
1	69.09	53.16	68.25	− 15.93	15.09	−0.84
2–3	71.38	55.18	75.73	−16.20	20.58	4.35
4–5	70.00	54.70	72.98	−15.30	18.28	2.98
6+	70.56	53.86	71.95	−16.70	18.09	1.39
**ANC** Visit						
Yes	62.65	54.63	73.49	−8.02	18.86	10.84
No	74.23	53.67	71.17	−20.56	17.54	−3.06
**Delivery Type**						
Vaginal	71.27	54.52	73.39	−16.27	18.87	2.12
CS	41.27	49.62	50.29	8.35	0.67	9.02
**Family size**						
< 5	69.18	54.75	72.66	−14.43	17.91	3.48
≥ 5	73.62	53.76	72.66	−19.86	18.90	−0.96
**Birth Interval**						
< 24 months	74.83	56.22	73.35	−18.61	17.13	−1.48
≥ 24 months	69.62	53.84	72.50	−15.78	18.66	2.88

### Decomposition analysis

#### Decomposition analysis of early breastfeeding initiation, 2005–2016

Overall, there have been increases in breastfeeding rates within 1 hour in Ethiopia from 2005 to 2016. The overall decomposition result suggested that the increases in breastfeeding rate within 1 hour has been explained by both the disparity in women’s characteristics and effects between the surveys. About half percentage of the increments in early initiation of breastfeeding rate was attributed to the differences in the composition of the respondents while half of the change in early initiation of breastfeeding change was due to the difference in the effect of the selected explanatory variable (Table 3).

**Table 3:** an overall decomposition analysis of change in breastfeeding rate over time in Ethiopia

EIBF	Coefficient	[95% Conf. Interval]	Pct
E	0.0905	(0.0753, 0.1058)	50.021***
C	0.0904	(0.0554, 0.1255)	49.979***
R	0.18097	(0.1494, 0.2125)***	

*E* endowment, *C* coefficient, *R* residual; ***: *p*-value < 0.01

In the detailed decomposition analysis, the overall increase in early initiation of breastfeeding rate from 2005 to 2016 was attributed to the difference in characteristics (endowment) of women between the survey. Covariates that provides a significant contribution to the improvements of EIBF were the mode of delivery, place of delivery, birth order, and size of child at birth. The increase in the composition of women with health facility delivery between 2005 and 2016 significantly contributed to the increase in early initiation of breastfeeding. Also, the change in composition of women with vaginal delivery significantly contributed to the increase in early initiation of breastfeeding rate over time. Similarly, an increased composition of women with first and 2nd-3rd birth order significantly contributed to the increase in early initiation of breastfeeding rate over the study period (2005–2016). In addition, the increase in women with small birth size between 2005 and 2016 significantly contributed to the increments of delayed initiation of breastfeeding (Table 4).

**Table 4:** Decomposition analysis in early initiation of breastfeeding among under-five children in the last ten years, Ethiopian Demographic and Health Survey 2005–2016

Variables	Difference due to characteristics (E)		Difference due to coefficient (C)	
	Coeff (95% CI)	Pct.	Coeff (95%CI)	Pct.
**Religion**				
*Catholic*	1	1	1	1
*Protestant*	.013167 (.006718, .019616)	7.276	.001421(−.0032222 .0060638)	.7851
*Muslim*	.025354 (.01345 .037257)	14.01	.012281(−.0040713 .028633)	6.7859
*Orthodox*	−.000876 (−.0019285 .00017133)	−.486	.031588 (.012072 .051104)	17.455
**Women education**				
No education	1	1	1	1
Primary	−.000731 (−.017629 .016166)	−.4041	.01188 (−.0062844 .030044)	6.5643
Secondary+	.000331 (−.0019283 .0025901)	.1828	−.00436 (−.022486 .013763)	−2.41
**Husband education**				
No education	1	1	1	1
Primary	.0138 (−.00046304 .027981)	7.603	.02594 (.0013799 .050506)*	14.335
Secondary+	−.00370(−.011594 .0042023)	− 2.042	.00926 (−.016928 .035451)	5.1175
**Birth order**				
1	1	1	1	1
2–3	.00553 (.00071964 .010344)**	3.057	.006751 (−.021646 .035148)	3.7303
4–5	.000618 (.00009254 .0011434)*	2.76	−.000402 (−.022471 .021667)	−.2220
6+	.0003701(−.00030126 .0010415)	.2045	.005199 (−.032211 .0426)	2.873
**Media exposure to EIBF**				
No	1	1	1	1
Yes	−.00789(−.016395 .00061322)	−4.3604	−.02667(−.052334–.0010006)	−14.736
**Wealth status**				
Poor	1	1	1	1
Middle	.000644(−.0038898 .0051767)	.356	−.00282 (−.020628 .014994)	− 1.5566
Rich	−.00246(−.013949 .0090282)	− 1.360	−.004635 (−.052111 .042842)	−2.5609
**Residence**				
Urban	1		1	1
Rural	−.011579(−.033564 .010407)	−6.398	−.01058(−.091644 .070478)	−5.848
**Place of delivery**				
Home	1	1	1	1
H/Institution	.035335 (.012752 .057918) ***	19.53	.006147(−.010381 .022674)	3.3965
**Type of delivery**				
Caesarian section	1	1	1	1
Vaginal	.081878 (.047254 .1165) ***	45.243	.17142 (.021396 .32144)* *	94.721
**Birth size**				
Large	1		1	1
Average	−.00380(−.0089242 .0013208)	−2.1007	.005543(−.025787 .036872)	3.0627
Small	−.0023 (−.0039578–.00064228)**	−1.2709	−.011915 (−.033867 .010037)	−6.584
**Trimester of ANC**				
1st trimester	1		1	
2nd trimester	−.003296 (−.01522 .0086289)	−1.821	−.0227 (−.061423 .016023)	−12.543
3rd trimester	.011497 (−.0063283 .029322)	6.3527	−.0288 (−.053146–.0046044) **	− 15.955
**Family size**				
< 5	1		1	
≥ 5	−.000543(−.0067437 .0056582)	−.2999	−.009255(−.043327 .024818)	−5.114
**Preceding Birth Interval**				
≤ 24 months	1		1	
> 24 months	.001574 (−.0096282 .012777)	.8698	−.00707 (−.082324 .068176)	−3.9091
**Sex of child**				
Male	1		1	
Female	.00142(−.0039387 .0067779)	.7844	.00481(−.023649 .03327)	2.6583
**Constants**			−.02722 (−.26075 .20631)	−15.041

*significant at 0.05

** significant at 0.01

*** significant at < 0.001

*Pct* percentage contribution

#### Difference due to effects of coefficient (C)

Half of the rise in early initiation of breastfeeding was attributed to behavioural changes to breastfeeding within 1 hour, controlling the effect of change in compositional characteristics. Covariates that have a significant on coefficient contribution to the observed change in breastfeeding within 1 hour are husband/partner education, mode of delivery, and trimester of antenatal care visit. Controlling all compositional change factors, 14% of breastfeeding within 1 hour increment was attributed to behaviour of husband/partner primary education over the last decade. Keeping the compositional factors constant, women who have attend late ANC was negatively correlated with the early initiation of breastfeeding over the last 10 years.

## Discussion

Delayed initiation of breastfeeding is a major public health issue in resource-limited settings like Ethiopia, the potential risk factor for neonatal mortality and morbidity [17, 23, 31, 32]. The incidence of breastfeeding within 1 hour in a community is a reflection of time to antenatal care, residence, and delivery service utilization [8, 11, 16, 18]. This study investigated the trends and determinants of early initiation of breastfeeding among under-five children in Ethiopia. The study aimed to identify the potential factors contributing to the change in the early initiation of breastfeeding either positively or negatively over the past 10 years in Ethiopia. The results of this study revealed that trends in early onset breastfeeding rates increased significantly between 2011 and 2016, even though they showed a decrease between 2005 and 2011. From 2005 to 2011, the breastfeeding rate decreased by 15.4/1000 births, while it increased by 17.6/1000 between 2011 and 2016, with an overall increase of 2.2/1000 from 2005 to 2016. In this study, we note that the timely initiation of breastfeeding from 2005 to 2016 was slightly lower than the WHO and UNICEFF Children’s Fund recommendations [33]. This may be due to Ethiopia’s government provide sufficient emphasis to key messages since 2004 on the timely introduction of breastfeeding [34] and establishing guidelines for feeding infants and young children.

From the decomposition analysis, the breastfeeding rate within an hour suggests an improvement over the survey period in Ethiopia. Hence, understanding the source of change has critical public health relevance to uncover what are contributing factors to the change in breastfeeding within an hour as well as understanding which factors are making progress in growing breastfeeding with an hour to assess current implementation strategies. The disparity in the composition of women over time was responsible for 50.02% of the rise in the breastfeeding rate within an hour over the entire sample survey period (2005–2016). An improvement in the composition of health facility distribution over the study showed a major impact on the increase in breastfeeding within 1 hour in accordance with other research performed in Tanzania [35], Bangladesh [36], South Asia [37], and Ethiopia [2, 19, 38]. This agreement confirms that prioritization should be given to encouraging and facilitating the use of health facility delivery services in order to make progress towards early initiation of breastfeeding. Hence delivering at a health facility reduces traditional beliefs about breastfeeding practice such as prelacteal feeding and misconception about colostrum because the health professionals empowered the mother to resist the role of external pressure and interference that promotes delayed initiation of breastfeeding. However, there is a suggestion that mothers who were delivered at home were highly exposed to practicing prelacteal feeding in Ethiopia [39–41]. Similarly vaginal delivery has a significant positive contribution to the rising of breastfeeding within 1 hour consistent with other studies conducted in Brazil [42], and Ethiopia [19, 21, 26, 43]. Since mothers who deliver vaginally have immediate skin-to-skin contact and infant treatment, as well as the onset of lactation is rapid that inhibits the likelihood of prelacteal feeding supplementation. However, a mother delivered by caesarean section typically associated with reducing timely initiation of breastfeeding hence the effect of anaesthesia delays the onset of lactation.

Also, increasing the composition of women with a child of small birth size has a significant contribution in reducing the timely initiation of breastfeeding within 1 hour of delivery. Since small babies may have an illness and stay away from their mother, resulting in the early supplementation of prelacteal feeding that inhibits the ability to initiate breastfeeding within 1 hour of delivery due to poor breastfeeding reflex and sucking inability similar findings were published in West Africa [20] and Zimbabwe [24], and prospective study in low and middle-income countries [1]. However, the large-size child can be considered as healthy and completely matured who possesses good reflection to initiate timely breastfeeding as viewed by their mothers and health care workers. This result indicates that the initiation of breastfeeding involves additional care and encouragement for mothers with small-sized infants.

Approximately 50% of early initiation breastfeeding progress was attributed to the shift in conduct towards breastfeeding initiation within 1 hour in Ethiopia, monitoring the effects of endowment characteristics. Factors substantially contributing to the positive and negative changes in early breastfeeding initiation were the achievement of husband/partner primary education and ANC visit at the third trimester respectively.

Increasing behavioural improvement in primary education for husbands/partners leads significantly to increasing early initiation of breastfeeding in Ethiopia, i.e. a 14 percent point change. Since husband education has a greater understanding regarding child care practice that receives information from health extension staff as well as daily mass media messages that can pressure their wife to start early breastfeeding even though there is no supporting evidence for this finding.

Late initiation of ANC visits i.e. at the 3rd trimester significantly contributed to the reduction of early initiation of breastfeeding by 16 percentage points. Since a mother attends ANC visit at the 3rd trimester after feeling uncomfortable and illness, they may not well aware of the importance of timely initiation of breastfeeding and colostrum to their child hence their first aim of attending ANC might be receiving treatment their health similar findings are published in low and middle-income countries [1] prospective study. Currently, focussed antenatal care guideline in Ethiopia recommends intensive health education about breastfeeding at the time of antenatal care visits for mothers.

### Strengths and limitations

One of the strengths of this analysis is that we used data from a national survey, the 2005–2016 EDHS. Therefore, the results of the study have thoughtful consequences at the personal, group and policy level.

However, there were limited sample sizes in some local areas represented in the survey, and the findings should therefore be viewed with caution. Since this study is a secondary data review of a national sample, other primary variables are not included, such as traditional attitudes, psycho-social influences, the preference of spouses for breastfeeding, and in-depth qualitative views of mothers. This research is focused on cross-sectional data and it is therefore difficult to show the cause and effect relationships of the covariates on the timely initiation of breastfeeding, and a recall bias may be susceptible to the survey responses.

## Conclusion

In this study, we find that the trends of early initiation of breastfeeding have increased marginally over the last 10 years in Ethiopia. With regarding to multivariate decomposition analysis, half of the overall rise in early initiation of breastfeeding was attributed to the change in compositional characteristics of women over 10 years in Ethiopia. Change in the composition of reproductive ages women’s characteristics according to place of delivery and mode of delivery were the major source of the increase in early breastfeeding initiation rate overtime while the size of the child at birth was contributed to delay timely breastfeeding over the study period.

Also half of the increase in early breastfeeding initiation rate was due to the changed behaviour of women towards early breastfeeding initiation over the last 10 years in Ethiopia. The change in behaviour of husband/partner education has a positive influence on the early initiation of breastfeeding while late initiation of antenatal care visits has been negatively correlated with early initiation of breastfeeding over time. Public action should be required, including encouraging the provision of women’s health facilities for more changes in the initiation of early breastfeeding. The ministry of health and other stakeholders should continue to enrich the coverage of early ANC visits and promoting community education regarding the need of early breastfeeding initiation to their newborns. Further interventions are needed among women deliver by caesarean section, one of the main barriers in delayed initiation of breastfeeding over the last 10 years in Ethiopia.

## Data Availability

The survey datasets used in this study was based on a publicly available dataset that is freely available online with no participant’s identity from. http://www.dhsprogram.com/data/available-datasets.cfm. Approval was sought from MEASURE DHS/ICF International and permission was granted for this use.

## Abbreviations

ANC: Antenatal care
CI: Confidence interval
DHS: Demographic health survey
EDHS: Ethiopian demographic and health survey
EIBF: Early initiation of breastfeeding
EAs: Enumeration areas
SNNP: Southern nations nationalities and peoples
UNICEF: United Nations international children’s fund
WHO: World health organization

## References

[1] Patel A, Bucher S, Pusdekar Y, Esamai F, Krebs NF, Goudar SS, Chomba E, Garces A, Pasha O, Saleem S, Kodkany BS, Liechty EA, Kodkany B, Derman RJ, Carlo WA, Hambidge KM, Goldenberg RL, Althabe F, Berrueta M, Moore JL, McClure EM, Koso-Thomas M, Hibberd PL (2015). Rates and determinants of early initiation of breastfeeding and exclusive breastfeeding at 42 days postnatal in six low and middle-income countries: a prospective cohort study. Reprod Health.

[2] Alebel A, Dejenu G, Mullu G, Abebe N, Gualu T, Eshetie S (2017). Timely initiation of breastfeeding and its association with birthplace in Ethiopia: a systematic review and meta-analysis. Int Breastfeed J.

[3] Debes AK, Kohli A, Walker N, Edmond K, Mullany LC (2013). Time to initiation of breastfeeding and neonatal mortality and morbidity: a systematic review. BMC Public Health.

[4] Nair N, Tripathy P, Prost A, Costello A, Osrin D (2010). Improving newborn survival in low-income countries: community-based approaches and lessons from South Asia. PLoS Med.

[5] Takahashi K, Ganchimeg T, Ota E, Vogel JP, Souza JP, Laopaiboon M, Castro CP, Jayaratne K, Ortiz-Panozo E, Lumbiganon P, Mori R (2017). Prevalence of early initiation of breastfeeding and determinants of delayed initiation of breastfeeding: secondary analysis of the WHO global Survey. Sci Rep.

[6] Arts M, Taqi I, Bégin F (2017). Improving the early initiation of breastfeeding: the WHO-UNICEF breastfeeding advocacy initiative. Breastfeed Med.

[7] World Health Organization. Guideline: protecting, promoting and supporting breastfeeding in facilities providing maternity and newborn services. 2017. 29565522

[8] Ndirangu M (2018). Trends and factors associated with early initiation of breastfeeding in Namibia: analysis of the demographic and health surveys 2000–2013. BMC Pregnancy Childbirth.

[9] Abie BM, Goshu YA (2019). Early initiation of breastfeeding and colostrum feeding among mothers of children aged less than 24 months in Debre Tabor, Northwest Ethiopia: a cross-sectional study. BMC Res Notes.

[10] Kiwango F (2020). Prevalence and factors associated with timely initiation of breastfeeding in the Kilimanjaro region, northern Tanzania: a cross-sectional study. BMC Pregnancy Childbirth.

[11] Lyellu HY (2020). Prevalence and factors associated with early initiation of breastfeeding among women in Moshi municipal, northern Tanzania. BMC Pregnancy Childbirth.

[12] Khan GN, Ariff S, Khan U, Habib A, Umer M, Suhag Z, Hussain I, Bhatti Z, Ullah A, Turab A, Khan AA, Garzon AC, Khan MI, Soofi S (2017). Determinants of infant and young child feeding practices by mothers in two rural districts of Sindh, Pakistan: a cross-sectional survey. Int Breastfeed J.

[13] Nkoka O, Ntenda PAM, Kanje V, Milanzi EB, Arora A (2019). Determinants of timely initiation of breast milk and exclusive breastfeeding in Malawi: a population-based cross-sectional study. Int Breastfeed J.

[14] Lawn J, Kerber K, Enweronu-Laryea C, Massee Bateman O (2009). Newborn survival in low resource settings—are we delivering?. BJOG Int J Obstet Gynaecol.

[15] Dewey KG, Vitta BS. Strategies for ensuring adequate nutrient intake for infants and young children during the period of complementary feeding. Washington: Alive & Thrive; 2013. 7.

[16] Derso T, Biks GA, Tariku A, Tebeje NB, Gizaw Z, Muchie KF, Shimeka A, Kebede Y, Abebe SM, Yitayal M, Ayele TA, Wubeshet M, Azmeraw T, Birku M, Fekadu A, Asrade G, Gebeyehu A, Tesfahun A, Alemu K (2017). Correlates of early neonatal feeding practice in Dabat HDSS site, Northwest Ethiopia. Int Breastfeed J.

[17] Victora CG, Bahl R, Barros AJD, França GVA, Horton S, Krasevec J, Murch S, Sankar MJ, Walker N, Rollins NC (2016). Breastfeeding in the 21st century: epidemiology, mechanisms, and lifelong effect. Lancet.

[18] Adhikari M, Khanal V, Karkee R, Gavidia T (2014). Factors associated with early initiation of breastfeeding among Nepalese mothers: further analysis of Nepal Demographic and health Survey, 2011. Int Breastfeed J.

[19] Belachew A (2019). Timely initiation of breastfeeding and associated factors among mothers of infants-age 0–6 months old in Bahir Dar City, northwest, Ethiopia, 2017: a community-based cross-sectional study. Int Breastfeed J.

[20] Ezeh OK (2019). Factors associated with the early initiation of breastfeeding in economic Community of West African States (ECOWAS). Nutrients.

[21] Gebremeskel SG, Gebru TT, Gebrehiwot BG, Meles HN, Tafere BB, Gebreslassie GW, Welay FT, Mengesha MB, Weldegeorges DA (2019). Early initiation of breastfeeding and associated factors among mothers of aged less than 12 months children in the rural eastern zone, Tigray, Ethiopia: a cross-sectional study. BMC Res Notes.

[22] Ekubay M, Berhe A, Yisma E (2018). Initiation of breastfeeding within one hour of birth among mothers with infants younger than or equal to 6 months of age attending public health institutions in Addis Ababa, Ethiopia. Int Breastfeed J.

[23] Smith ER, et al. Delayed breastfeeding initiation and infant survival: A systematic review and meta-analysis. PloS one. 2017;12(7):e0180722. 10.1371/journal.pone.0180722 PMC5528898 28746353

[24] Mukora-Mutseyekwa F (2019). Predictors of early initiation of breastfeeding among Zimbabwean women: secondary analysis of ZDHS 2015. Matern Health Neonatol Perinatol.

[25] Setegn T, Gerbaba M, Belachew T (2011). Determinants of timely initiation of breastfeeding among mothers in Goba Woreda, south East Ethiopia: a cross-sectional study. BMC Public Health.

[26] Tewabe T (2016). Timely initiation of breastfeeding and associated factors among mothers in Motta town, east Gojjam zone, Amhara regional state, Ethiopia, 2015: a cross-sectional study. BMC Pregnancy Childbirth.

[27] Demographic, C.E (2011). Health Survey 2011 Addis Ababa, Ethiopia, and Calverton.

[28] Demographic, E., Health Survey 2005 (2011). Addis Ababa, Ethiopia, and Calverton.

[29] CSACE, I (2016). Ethiopia demographic and health survey 2016.

[30] Powers DA, Yoshioka H, Yun M-S (2011). Mvdcmp: multivariate decomposition for nonlinear response models. Stata J.

[31] Karimi FZ, Sadeghi R, Maleki-Saghooni N, Khadivzadeh T (2019). The effect of mother-infant skin to skin contact on success and duration of first breastfeeding: a systematic review and meta-analysis. Taiwan J Obstet Gynecol.

[32] Bhandari S, Thorne-Lyman AL, Shrestha B, Neupane S, Nonyane BAS, Manohar S, Klemm RDW, West KP (2019). Determinants of infant breastfeeding practices in Nepal: a national study. Int Breastfeed J.

[33] Organization, W.H. Evidence for the ten steps to successful breastfeeding: World Health Organization; 1998.

[34] World Health Organization. Implementing the Global Strategy for Infant and Young Child Feeding: Geneva, 3-5 February 2003: meeting report. 2003.

[35] Victor R, et al. Determinants of breastfeeding indicators among children less than 24 months of age in Tanzania: a secondary analysis of the 2010 Tanzania Demographic and health Survey. BMJ Open. 2013;3(1). 10.1136/bmjopen-2012-001529 PMC3549262 23299109

[36] Karim F, Billah SM, Chowdhury MAK, Zaka N, Manu A, Arifeen SE, Khan ANS (2018). Initiation of breastfeeding within one hour of birth and its determinants among normal vaginal deliveries at primary and secondary health facilities in Bangladesh: a case-observation study. PLoS One.

[37] Sharma IK, Byrne A (2016). Early initiation of breastfeeding: a systematic literature review of factors and barriers in South Asia. Int Breastfeed J.

[38] Temesgen H, Negesse A, Woyraw W, Getaneh T, Yigizaw M (2018). Prelacteal feeding and associated factors in Ethiopia: systematic review and meta-analysis. Int Breastfeed J.

[39] Tariku A, Biks GA, Wassie MM, Gebeyehu A, Getie AA (2016). Factors associated with prelacteal feeding in the rural population of Northwest Ethiopia: a community cross-sectional study. Int Breastfeed J.

[40] Legesse M, Demena M, Mesfin F, Haile D (2014). Prelacteal feeding practices and associated factors among mothers of children aged less than 24 months in Raya kobo district, north eastern Ethiopia: a cross-sectional study. Int Breastfeed J.

[41] Amele EA (2019). Prelacteal feeding practice and its associated factors among mothers of children age less than 24 months old in southern Ethiopia. Ital J Pediatr.

[42] Vieira TO, Vieira GO, Giugliani ERJ, Mendes CMC, Martins CC, Silva LR (2010). Determinants of breastfeeding initiation within the first hour of life in a Brazilian population: a cross-sectional study. BMC Public Health.

[43] Liben ML, Yesuf EM. Determinants of early initiation of breastfeeding in Amibara district, Northeastern Ethiopia: a community based cross-sectional study. Int Breastfeed J. 2016;11(1):1–7. 10.1186/s13006-016-0067-8 PMC4826535 27064535

